# Adsorption therapy in critically ill with septic shock and acute kidney injury: a retrospective and prospective cohort study

**DOI:** 10.1186/s13613-020-00772-7

**Published:** 2020-11-18

**Authors:** Gregor A. Schittek, Philipp Zoidl, Michael Eichinger, Simon Orlob, Holger Simonis, Martin Rief, Philipp Metnitz, Tobias Fellinger, Jens Soukup

**Affiliations:** 1 Department of Anaesthesiology and Intensive Care Medicine, Division of General Anaesthesiology and Intensive Care Medicine, Medical University of Graz, Auenbruggerplatz 5, 8036 Graz, Austria; 2 Austrian Centre for Documentation and Quality Assurance in Intensive Care, Vienna, Austria; 3 Department of Anaesthesiology, Intensive and Palliative Care, Carl-Thiem-Hospital Cottbus, Cottbus, Germany

**Keywords:** Cytosorb, Haemoadsorption, Mortality, Outcome, Sepsis, Septic shock, Acute kidney injury

## Abstract

**Background:**

Haemoadsorption has been described as an effective way to control increased pro- and anti-inflammatory mediators (“cytokine storm”) in septic shock patients. No prospective or randomised clinical study has yet confirmed these results. However, no study has yet prospectively specifically investigated patients in severe septic shock with sepsis-associated acute kidney injury (SA-AKI). Therefore, we aimed to examine whether haemoadsorption could influence intensive care unit (ICU) and hospital mortality in these patients. Furthermore, we examined the influence of haemoadsorption on length of stay in the ICU and therapeutic support.

**Methods:**

Retrospective control group and prospective intervention group design in a tertiary hospital in central Europe (Germany). Intervention was the implementation of haemoadsorption for patients in septic shock with SA-AKI. 76 patients were included in this analysis.

**Results:**

Severity of illness as depicted by APACHE II was higher in patients treated with haemoadsorption. Risk-adjusted ICU mortality rates (O/E ratios) did not differ significantly between the groups (0.80 vs. 0.83). We observed in patients treated with haemoadsorption a shorter LOS and shorter therapeutic support such as catecholamine dependency and duration of RRT. However, in multivariate analysis (logistic regression for mortality, competing risk for LOS), we found no significant differences between the two groups.

**Conclusions:**

The implementation of haemoadsorption for patients in septic shock with acute renal failure did not lead to a reduction in ICU or hospital mortality rates. Despite univariate analysis delivering some evidence for a shorter duration of ICU-related treatments in the haemoadsorption group, these results did not remain significant in multivariate analysis.

*Trial registration* CytoSorb^®^ registry https://clinicaltrials.gov/ct2/show/NCT02312024. December 9, 2014. Database: https://www.cytosorb-registry.org/ (registration for content acquisition is necessary)

## Introduction

Sepsis is a life-threatening organ dysfunction caused by a dysregulated immune response after infection and is one of the worldwide leading causes of death [1, 2]. The definition of septic shock, as a subtype of sepsis, includes circulatory instability and therefore the need for vasopressors after volume substitution as well as cellular and metabolic malfunctioning with an increased lactate level. Septic shock is associated with higher in-hospital mortality compared to patients that suffer from sepsis alone. Among septic shock patients, those with acute renal failure (ARF) have the highest mortality [3]. Presumably, a “cytokine storm” causes the worsening of sepsis to septic shock [4]. This hyperinflammatory reaction is not only induced by the release of endogenous cytokines (IL-1β, IL-6, IL-12, TNF-α), but also the exotoxic ones, like pattern-associated molecular patterns (PAMPs) or damage-associated molecular pattern (DAMPs).

Whole blood absorbers can remove excessive inflammatory mediators like cytokines, chemokines, haemoglobin, myoglobin, bilirubin, bile acids, bioactive lipids, light chains from antibodies, toxins, enterotoxins (Shiga toxins, alpha-haemolysin, gangrene toxins, diphtheria toxins) and other toxic metabolites [5]. The absorber can be connected to either an extracorporeal membrane oxygenation (ECMO) device or renal replacement therapy (RRT) and will partly eliminate the abovementioned mediators [6]. With regard to the current literature on haemoadsorption, it would seem reasonable and associated only with the slightest logistics to add Cytosorb™ to the RRT in septic patients with ARF for haemoadsorption. However, no study has yet prospectively analysed patients in septic shock with sepsis-associated acute kidney injury (SA-AKI) [6–19]. Until now, only case reports/series and retrospective studies have supported in their findings the utilisation of the absorber we used in this study [6–20]. While the adsorber has not been approved by the FDA but for COVID-19 emergency treatment, the European Medicines Agency (EMA) decided to do so. Despite its approval in Europe in 2011, there still have not been any prospective studies supporting the efficacy of the absorbers. Only one randomised, controlled multicentre trial exists. In that study, the primary endpoint was a reduction in IL-6 in sepsis via absorption. Secondary endpoints were a reduction in the multiorgan failure (MOF) rate and ventilator-free days and a positive impact on oxygenation. The primary and secondary endpoints of this multicentre trial showed no impact of haemoadsorption. However, patients in severe sepsis with ARF were only a small subgroup (*N* = 15 [32%] vs. *N* = 8 [16%]), so no analysis of patients in septic shock with ARF was possible [8].

One recent randomised-controlled single-centre pilot study described 20 patients in septic shock without ARF and showed a significant reduction in catecholamine requirements as well as PCT levels in patients who received therapy with the absorber [7]. The authors could not conduct an outcome analysis because of the small patient number.

Due to the missing evidence regarding haemoadsorption, we decided in 2015 to participate in the CytoSorb^®^ registry trial and further conduct a pre–post analysis. The objective of our analysis was to determine whether the implementation of haemoadsorption in septic shock patients with SA-AKI influences intensive care unit (ICU)-related outcomes. Primarily, we aimed to examine whether haemoadsorption would influence ICU mortality. Secondarily, we aimed to examine the influence on length of treatment, such as renal replacement therapy (RRT), ICU length of stay (LOS), catecholamines and duration of ventilatory support.

## Materials and methods

### Selection and description of participants

After obtaining approval for participation in the Cytosorb^®^ registry by the local ethics committee [AS 88(bB)/28.01.2015, Chairman Prof. M. Matthias], we prospectively collected data in our ICU at a tertiary hospital in Cottbus, Germany, starting from 2015, for a period of 3 years [20]. We decided to utilise haemoadsorption with Cytosorb™ in all patients in the aforementioned period with following condition: septic shock (rising noradrenaline dose above 20 µg/min.) with sepsis-associated acute kidney injury in adult patients treated in our ICU. The Cytosorb^®^ registry was designed to explore the use of the Cytosorb™ (Cytosorbents Corp., NJ, USA) absorber in critically ill patients under real-life conditions using all relevant information (e. g., diagnosis, comorbidities, treatment/concomitant medication, clinical laboratory parameters, outcome) during haemoadsorption (ClinicalTrials.gov Identifier: NCT02312024) [20, 21]. The primary endpoint of this prospective, multicentre registry involving more than 130 centres from 22 countries was to compare the in-hospital mortality predicted according to the APACHE II score. APACHE II was calculated for the 24 h prior to initiation of CVVHDF/haemoadsorption (according to the registry protocol). Additionally, we retrospectively analysed identified patients’ group from our ICU treated from 01/2012 to 12/2013 with identical clinical inclusion criteria as a historical control group with approval from the local ethics committee. In detail, inclusion criteria were septic shock (rising noradrenaline dose above 20 µg/min.) with sepsis-associated acute kidney injury in adult patients treated in our ICU. Due to reconstruction measures and the used patient data management systems, we could only extend the historic control group back to 2012. The 3 years of prospective data recruitment were by default from the study protocol of the Cytosorb^®^ registry. Thus our study was a single-centre prospective intervention study with historical controls.

### Treatment protocol and material

All patients were treated in accordance with the local sepsis treatment protocol, which was based on the actual guidelines valid during each treatment period [22, 23]. We evaluated the adequacy of fluid resuscitation therapy or vasopressor support using pulse indicated continuous cardiac output (PiCCO^®^, Getinge Deutschland GmbH, Rastatt, Germany) or ultrasound. The initial calculated antimicrobial therapy based on the assumed or underlying septic focus according the actual hospital recommendations. The continuous veno-venous haemodiafiltration (CVVHDF, Prismaflex, M150 Filter, Fa. Baxter International Inc.) with or without haemoadsorption was used in all patients with septic shock with acute renal failure (failure stage of RIFLE criteria for 12–24 h, comparative to KDIGO stage 3) according to the RIFLE classification [24], increasing catecholamine dependency above a noradrenaline dose of 20 µg/min (despite adequate fluid resuscitation therapy) and elevated IL-6 higher than 500 pg/ml. All patients were anticoagulated via the continuous administration of heparin. Thus, all patients included in our analysis presented with septic shock, an increasing catecholamine dependency above a noradrenaline dose of 20 µg/min and were treated with CVVHDF due to ARF.

Absorbers were changed when the CVVHDF system had to be changed or further adsorption therapy was needed. The further need for adsorption therapy was assessed whether the patient became increasingly unstable again after termination of the adsorption therapy or interleukins started increasing again.

### Data acquisition and statistical analysis

Starting in January 2015, we prospectively acquired data for the Cytosorb^®^ registry. According to the underlying protocol of the Cytosorb^®^ registry, we chose ICU mortality as primary endpoint for our evaluation as well. Secondarily, we evaluated the hospital mortality and length of treatments in days (ICU/hospital/ventilatory support/catecholamine therapy/CVVHDF).

Data analysis and statistics were performed with IBM SPSS Statistics 25. Data are described with median or mean, as necessary. The interquartile range (IQR) or 95% confidence interval is displayed in parentheses as appropriate. Because of the small sample size, all tests were performed as exact tests. To assess group differences, we used Levene’s test and the Mann–Whitney *U* test. We used *α* = 5%. Missing data regarded only laboratory test results, and therefore did not influence the interpretation of primary or secondary results. Significant results and differences are marked with an asterisk (*) in the tables. For the purposes of this article, the term “significant” is used when data reached statistical significance, defined as *p* < 0.05. Because of the missing information in the literature, no power analysis could be performed initially. Therefore, we decided to participate in the CytoSorb^®^ registry according to protocol. The control group size was also not calculated, but we screened and evaluated all patients since the implementation of our patient data management system in 2012 until the beginning of the Cytosorb™ implementation in 2014. To enable comparison between the groups, we calculated the observed-to-expected (ICU and hospital) mortality ratio (O/E ratio). Further analyses included logistic regression of ICU mortality and hospital mortality adjusted with the covariates "haemoadsorption", "APACHE II", "PCT prior to CVVHDF", "CRP prior to CVVHDF " and "catecholamine dosage before initiation of CVVHDF". The same covariates were used in a Fine and Gray model for the subdistribution hazard ratio of the event of "alive discharge from ICU" in presence of the competing event of death in the ICU.

## Results

From 1/2015 to 5/2018, a total of 2,102 patients with septic shock were treated in our ICU. Of these, 844 patients developed ARF and 159 were treated with CVVHDF. We identified 43 patients fulfilling the inclusion criteria. In the historic control group (from 1/2012 to 12/2013), 672 patients with septic shock were treated in our ICU; 164 developed ARF and 70 of them were treated with CVVHDF. Furthermore, we identified 33 patients for the control group fulfilling the inclusion criteria. The characteristics of our patients (at the moment of initiation of CVVHDF or haemoadsorption) before and after the implementation of haemoadsorption were similar (see Table 1). The only difference between the groups was the significantly higher severity of illness as depicted by APACHE II (*p* = 0.008) and catecholamine dependency in patients treated with haemoadsorption (*p* = 0.001) (Table 1).

**Table 1:** Demographics of the study population (control group vs haemoadsorption therapy)

Characteristic	Control group (*n* = 33)	Haemoadsorption group (*n* = 43)	*p* value
Age (years)	62 (53, 74)	63 (52, 71)	NS
Sex female/male	4/29	12/31	NS
Weight (kg)	87 (78, 105)	90 (75, 100)	NS
Septic focus			NS
Abdominal	9	13	
Catheter infection	0	1	
CNS	1	0	
Pulmonary	16	13	
Soft tissue	2	7	
Unclear	5	5	
Urinary tract	0	4	
CRP before CVVHDF (mg/l) (*n* = 33 vs 42)	253 (173, 363)	250 (167, 337)	NS
PCT before CVVHDF (ng/l)	7 (2, 30)	28 (11, 67)	0.002
IL-6 before CVVHDF (pg/ml) (*n* = 0 vs 41)	Not measured	5000 (939, 5000)	
Noradrenaline before CVVHDF (µg/min)	44 (38, 62)	64 (48, 90)	0.005
Hydrocortisone administration	82%	81%	NS
APACHE II before CVVHDF initiation	35 (33, 40)	39 (36, 42)	0.01
Lowest MAP 24 h before CVVHDF	65 (57, 73)	53 (40, 65)	0.03
Lowest GCS 24 h before CVVHDF (estimated if patient already sedated)	11 (3, 15)	3 (3, 3)	NS

*CNS* central nervous system, *CRP* C-reactive protein, *PCT* procalcitonin, *IL-6* interleukins 6, *CVVHDF* continuous veno-venous haemodiafiltration, *MAP* middle arterial blood pressure, *GCS* Glasgow Coma Scale, *NS* not significant

The observed ICU mortality rates of 72.1% (95% CI 58.7–85.5%) vs. 66.7% (95% CI 52.6–80.8%) were not significantly different between the groups (Table 2). When comparing risk-adjusted hospital mortality rates (observed-to-expected mortality ratios, short: O/E ratios), no significant difference between the groups was found: 0.88 (95% CI 0.77–0.99) vs. 0.80 (95% CI 0.65–0.94). With regard to the use of haemoadsorption cartridges in survivors, approximately one cartridge per patient was utilised as the median (IQR 1, 2) for 35.5 h (17, 47).

**Table 2:** Treatment and outcome of control and HA group (Cytosorb™-group)

Outcome measure	Control group (*N* = 33)	Cytosorb™-group (*N* = 43)	*p* value
ICU mortality rate	66.7% (22/33)	72.1% (31/43)	
O/E ratio ICU mortality	0.81 (0.65–0.94)	0.82 (0.72–0.93)	
Hospital mortality rate	66.7% (22/33)	76.7% (33/43)	
O/E ratio hospital-mortality	0.81 (0.65–0.94)	0.88 (0.77–0.99)	
LOS ICU (days [d])	21 (6, 54)	12 (3, 23)	0.026
LOS ICU for survivors (d) [*N* = 11 vs 12]	64 (45, 80)	29 (20, 40)	0.006
LOS ICU for non-survivors (d), [*N* = 22 vs 31]	10 (4, 20)	7 (3, 14)	0.115
LOS hospital (d)	25 (11,71)	15 (5,30)	0.040
LOS hospital survivors (d)	86 (68, 122)	52 (38, 45)	0.004
LOS hospital non-survivors (d)	13 (5, 28)	8 (5, 17)	0.096
Length of ventilatory support (d)	19 (5, 41)	8 (3,17)	0.009
Length of ventilatory support for survivors (d)	42 (37, 72)	20 (11, 26)	0.001
Length of ventilatory support non-survivors (d)	10 (4, 20)	6 (3, 13)	0.121
Length of CVVHDF (d)	6 (3, 16)	3 (2, 7)	0.012
Length of CVVHDF survivors (d)	12 (4, 19)	6 (3, 10)	0.260
Length of CVVHDF non-survivors (d)	4 (2, 10)	2 (2, 4)	0.047
Length of catecholamine administration (d)	18 (7, 30)	8 (3, 13)	0.001
Length of catecholamine administration survivors (d)	29 (21, 47)	10 (7, 12)	> 0.001
Length of catecholamine administration non-survivors (d)	11 (4, 20)	7 (3, 13)	0.043

*ICU* intensive care unit, *CVVHDF* continuous veno-venous haemodiafiltration

We further analysed ICU and hospital mortality by multivariate analysis (logistic regression, Additional file 1: Table S1). In both models we adjusted mortality with the covariates “haemoadsorption", "APACHE II", "PCT prior to CVVHDF", "CRP prior to CVVHDF " and "catecholamine dosage before initiation of CVVHDF". The coefficient for haemoadsorption was not significant in either of those models (OR ICU 0.3–3.0 [*p* = 0.99] and OR hospital 0.43–4.05 [*p* = 0.64]).

**Fig. 1:**
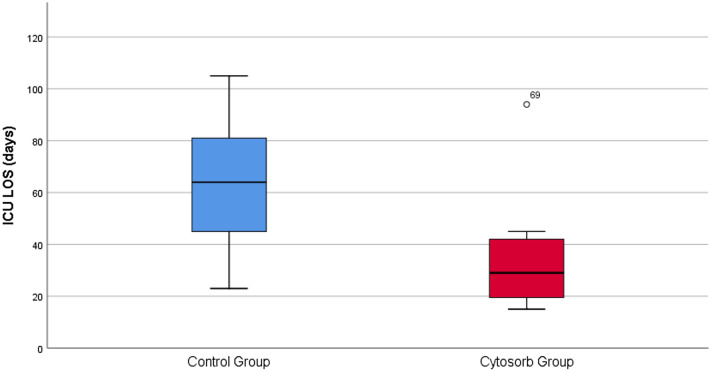
Blue: control group—patients without haemoadsorption (*n* = 11); red: patients with haemoadsorption (*n* = 12)

**Fig. 2:**
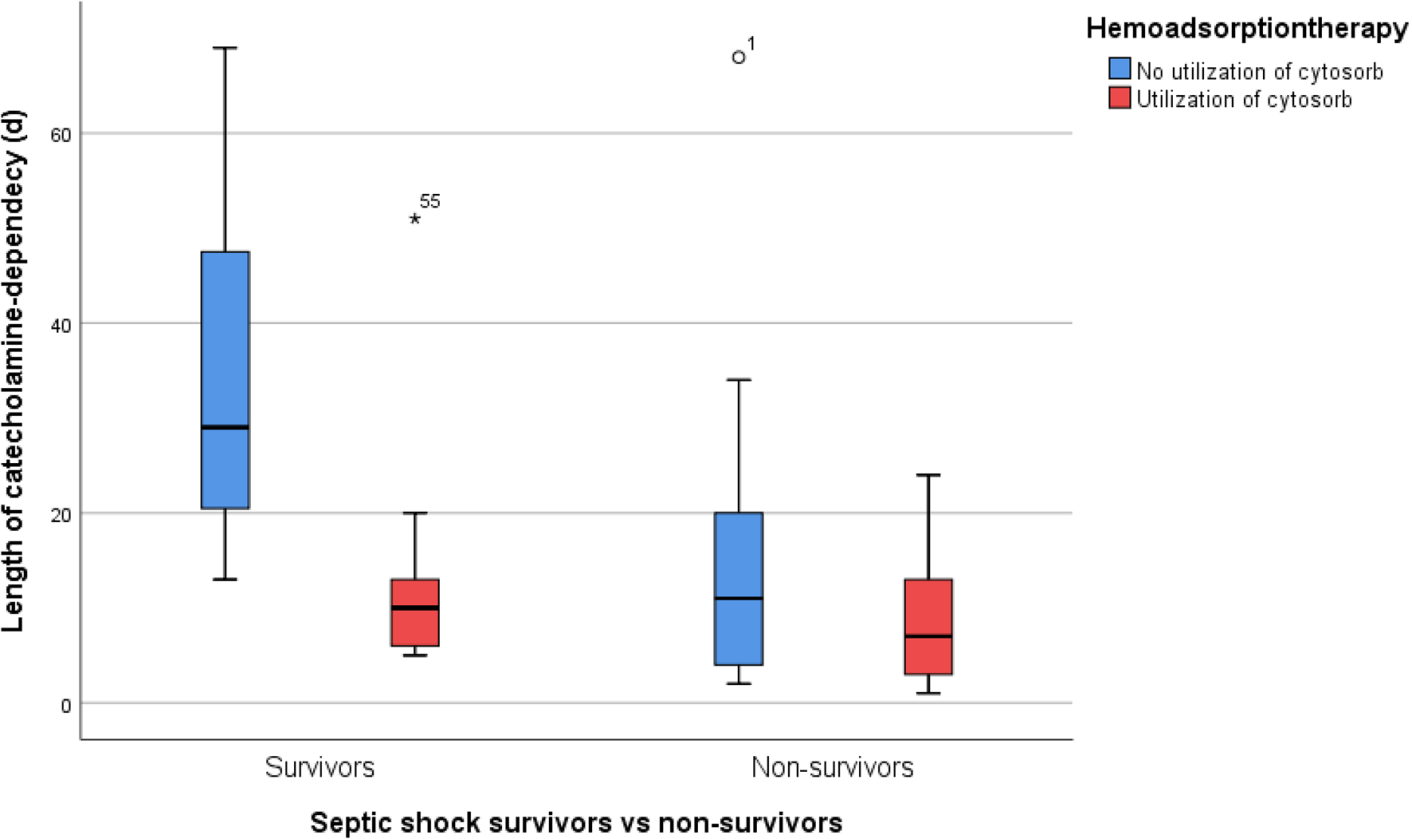
ICU length of catecholamine dependency for patients without (blue) and with (red) haemoadsorption. For ICU survivors, *N* = 11 (control group) vs. *N* = 12 (Cytosorb™ group). For ICU non-survivors, *N* = 22 vs. *N* = 31, respectively

In univariate analysis of all patients, we found that LOS, ventilatory support, duration of CVVHDF and duration of catecholamine administration were significantly lower for patients in the haemoadsorption group (*p* < 0.01) (Table 2). These results remained significant when only survivors were analysed. We found that length of stay and treatments except for duration of CVVHDF were in favour of the haemoadsorption group (Table 2, Figs. 1, 2). Their ICU-LOS for survivors was 29 (20, 40) vs. 64 (45, 80) days (*p* = 0.006).

**Fig. 3:**
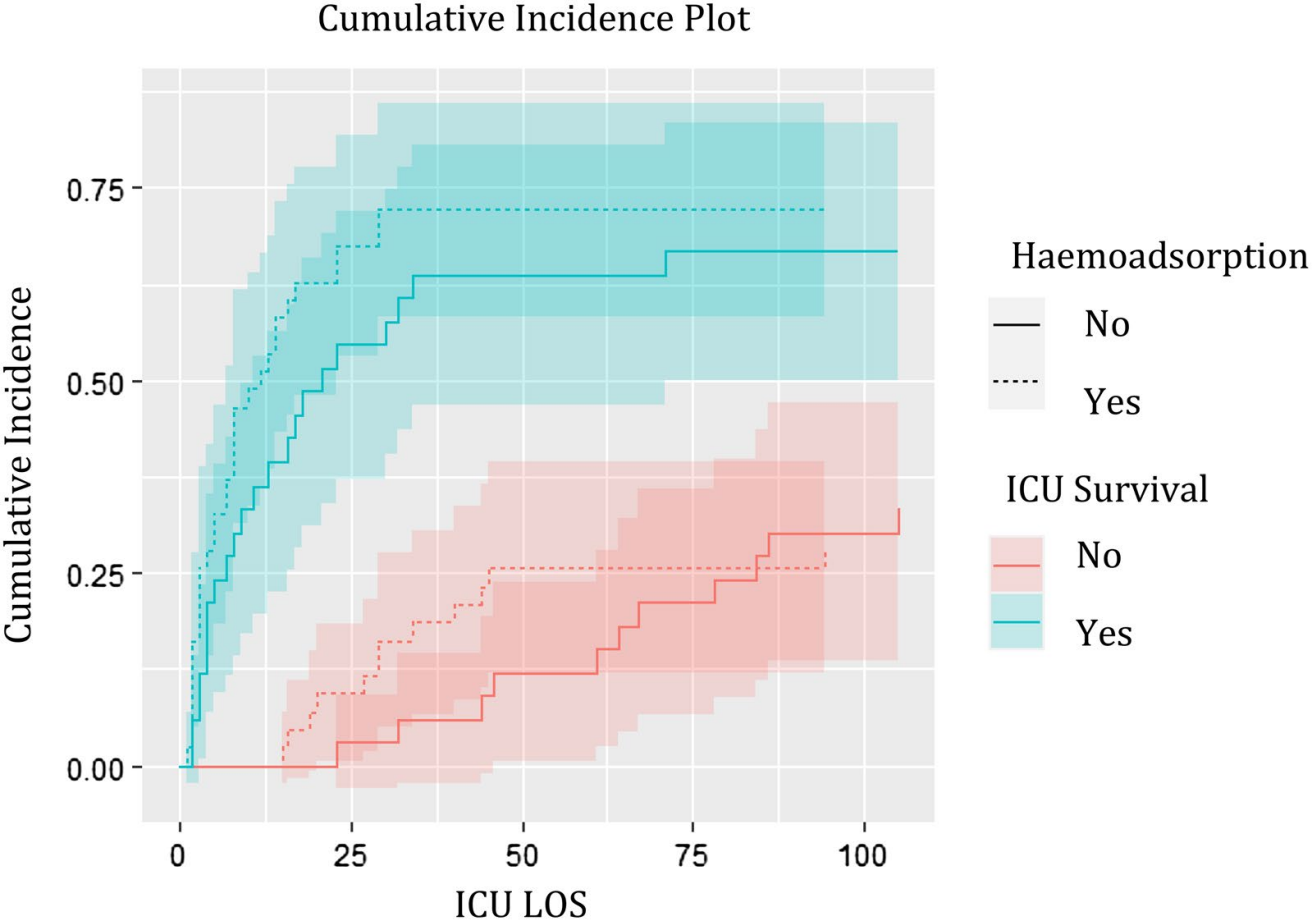
Influence of haemoadsorption on the time to event for alive discharge from ICU vs. death in ICU unadjusted cumulative incidence plots. The confidence intervals overlap, showing no significant difference in the cumulative incidences

However, when analysing LOS for all patients with a competing risk model (Fine and Gray), where LOS was adjusted for the same covariates as mentioned for the logistic regression above, no difference between the two groups remained significant (Fig. 3).

## Discussion

Our study shows that after adjustment for covariates, there was no benefit of haemoadsorption in patients with septic shock on either mortality or LOS in the ICU. Regarding LOS, we found a significant difference between the two groups only in univariate analysis, but not in multivariate analysis. It cannot be excluded that this discrepancy might be due to the small number of observations, which makes adjustment for several covariates difficult.

Although we found some evidence, that application of haemoadsorption reduced the duration of several invasive ICU-related treatments, such as RRT, ventilation and catecholamine dependency; it has to be highlighted, that despite the shorter duration, these patients did not show lower ICU or hospital mortality. Our findings are in line with the existing RCTs, where the authors stated that no mortality reduction could be found [7, 8]. In the multicentric RCT form Schädler et al. patients with sepsis and ARDS were investigated. However, the investigated groups had far lower ARF rates (16% vs. 32%) and APACHE II values (23–25 vs. 35–39) than our patients. Despite our matching resulting in more homogenous groups, we could only confirm their main result regarding mortality [8]. In the second RCT from Hawchar et al., only 20 patients without ARF were investigated and thus no mortality analysis was planned. In September 2019, a retrospective study assessing septic shock patients and CVVHDF was published [19]. In this publication, the authors stated that they observed a non-significant reduction in 28-day mortality (48% vs. 51%). After statistically adjusting their data, they found that patients treated with haemoadsorption had significantly lower 28-day mortality (52% vs. 72%). Comparison of their results with our own or further studies is difficult due to the retrospective nature, lack of randomisation in treatment assignment and missing explanations as to why each treatment was chosen.

Regarding secondary outcome analyses, we observed that after adjustment of covariates, patients with haemoadsorption did not have lower mortality rates. While Schädler et al. [8] did not evaluate treatment duration (ICU, hospital, ventilator therapy, CVVHDF), Hawchar et al. could also not find a significant difference regarding ICU-LOS in their pilot trial [7]. While the authors described no effect on mortality or LOS, they stated that that they could significantly reduce catecholamines during haemoadsorption [7]. Although these results seem to be in line with ours, their described result is only referring to the pre–post values of the haemoadsorption group and is not comparing the two groups to each other. Further, because their control group was older and had higher expected mortality (57% vs. 70%) their results could just be the description of the effect of convalescence of a healthier population group, or merely be coincidence due to the small sample size.

Furthermore, we noticed that a reduction in the negative effects of the cytokine storm has also been described as an immunomodulatory effect by macrolides. In 2013, it was shown in an RCT with 600 septic patients in Greece that the i.v. administration of clarithromycin (1 g daily over 4 days) reduced the treatment costs by approximately 30% [29]. In the subgroup analysis of patients with septic shock and multiorgan failure, the authors found that patients treated with additional clarithromycin had significantly lower 28-day mortality (73.1% [19/26] vs. 53.6% [15/28]) and shorter duration of sepsis treatment (10 vs. 6 days). Since these results are derived of a subgroup of < 10% of the total study population, they have also to be interpreted as exploratory and with caution.

The difference in the severity of illness between our two investigated groups did not introduce a bias since we compare only risk-adjusted mortality rates. However, we have to admit that APACHE II was not calculated using the worst values of the first 24 h after admission. We used instead the last 24 h before administration of haemoadsorption to calculate APACHE II values according to the registry’s protocol. However, since this was done for both groups in the same way, values still remain comparable.

In addition, it has to be mentioned that although haemoadsorption was not to be considered as ultima ratio for the patients in our setting, the availability of this new technology may have encouraged clinicians to further escalate sepsis therapy for patients, which would otherwise not have been performed (e.g. CVVHDF). This would explain why more patients in the haemoadsorption group had lower MAP and GCS scores.

A limitation of our study is of course its monocentric design. This could have caused a selection bias in the study population and how the data were acquired, which may have led to results that cannot be generalised. In this context, an RCT with the primary endpoint of a reduction in ICU-related treatment duration (possibly through immunomodulation) could form a base for a further evaluation of haemoadsorption in septic shock and its efficacy as part of a sepsis treatment bundle.

## Conclusions

The implementation of haemoadsorption for patients in severe septic shock with acute renal failure did not lead to a reduction in ICU or hospital mortality rates. Despite univariate analysis delivering some evidence for a shorter duration of ICU-related treatments in the haemoadsorption group, these did not remain significant in multivariate analysis. Further studies are needed to evaluate the effects of haemoadsorption in critically ill patients with severe septic shock and SA-AKI.

## Supplementary information

**Additional file 1: Table S1.** Logistic regression on mortality and Fine & Gray Model on LOS. The logistic regression model with ICU mortality and hospital mortality respectively were fitted with the covariates "haemoadsorption", "APACHE II", "PCT prior to CVVHDF", "CRP prior to CVVHDF " and "catecholamine dosage before initiation of CVVHDF".. The coefficient for haemoadsorption was not significant in neither of those models. A Fine and Gray model for the subdistribution hazard ratio of the event of alive discharge from ICU in presence of the competing event of death in the ICU was fitted with the same covariates as the logistic regression models. None of the covariates showed a significant influence.

## Data Availability

On demand and additionally via the Cytosorb™ register.

## Abbreviations

APACHE II: Acute Physiology and Chronic Health Evaluation
ARF: Acute renal failure
CRP: C-reactive protein
CVVHDF: Continuous veno-venous haemodiafiltration
DAMPs: Damage-associated molecular pattern
ECMO: Extracorporeal membrane oxygenation
EMA: European Medicines Agency
FDA: Food and Drug Administration
GCS: Glasgow Coma Scale
HA: Haemoadsorption
HR: Hazard ratio
ICU: Intensive care unit
LOS: Length of stay
MAP: Mean arterial blood pressure
MOF: Multiorgan failure
O/E: Observed to expected
OR: Odds ratio
PAMPs: Pattern-associated molecular patterns
PCT: Procalcitonin
RCT: Randomised controlled trial
RRT: Renal replacement therapy
SA-AKI: Sepsis associated acute kidney injury
