# Supplementary material for: Adsorption therapy in critically ill with septic shock and acute kidney injury: a retrospective and prospective cohort study
Source: Ann Intensive Care. 2020 Nov 18;10:154. doi: 10.1186/s13613-020-00772-7 (PMC7672170; doi:10.1186/s13613-020-00772-7)
Supplement: Supplementary file 1 — Additional file 1: Table S1. Logistic regression on mortality and Fine & Gray Model on LOS. The logistic regression model with ICU mortality and hospital mortality respectively were fitted with the covariates "haemoadsorption", "APACHE II", "PCT prior to CVVHDF", "CRP prior to CVVHDF " and "catecholamine dosage before initiation of CVVHDF".. The coefficient for haemoadsorption was not significant in neither of those models. A Fine and Gray model for the subdistribution hazard ratio of the event of alive discharge from ICU in presence of the competing event of death in the ICU was fitted with the same covariates as the logistic regression models. None of the covariates showed a significant influence. [file 13613_2020_772_MOESM1_ESM.docx]

Additional Table S1: Logistic regression on mortality and Fine & Gray Model on LOS. The logistic regression model with ICU mortality and hospital mortality respectively were fitted with the covariates "haemoadsorption", "APACHE II", "PCT prior to CVVHDF", "CRP prior to CVVHDF " and "catecholamine dosage before initiation of CVVHDF".. The coefficient for haemoadsorption was not significant in neither of those models. A Fine and Gray model for the subdistribution hazard ratio of the event of alive discharge from ICU in presence of the competing event of death in the ICU was fitted with the same covariates as the logistic regression models. None of the covariates showed a significant influence.

| ICU Mortality (logistic regression) | Coefficient | OR | OR lower | OR upper | p-value |
| --- | --- | --- | --- | --- | --- |
|  | (Intercept) | 2.22 | 0.04143 | 119 | 0.6908 |
|  | Haemoadsorption | 1.003 | 0.3354 | 2.971 | 0.9959 |
|  | APACHE II | 0.9963 | 0.8966 | 1.109 | 0.9441 |
|  | CRP | 0.9974 | 0.9934 | 1.001 | 0.193 |
|  | PCT | 0.9974 | 0.9877 | 1.005 | 0.5354 |
|  | Noradrenaline | 1.017 | 0.9954 | 1.042 | 0.1544 |
| Hospital Mortality (logistic regression) | Coefficient | OR | OR lower | OR upper | p-value |
|  | (Intercept) | 3.794 | 0.0612 | 249.6 | 0.5247 |
|  | Haemoadsorption | 1.307 | 0.4256 | 4.054 | 0.6383 |
|  | APACHE II | 0.9725 | 0.8703 | 1.086 | 0.6169 |
|  | CRP | 0.9979 | 0.9938 | 1.002 | 0.2923 |
|  | PCT | 0.9974 | 0.9875 | 1.005 | 0.5407 |
|  | Noradrenaline | 1.021 | 0.9985 | 1.049 | 0.09199 |
| LOS (Fine and Gray Model) | Coefficient | HR | HR lower | HR upper | p-value |
|  | Haemoadsorption | 1.137 | 0.4849 | 2.666 | 0.7677 |
|  | APACHE II | 1.005 | 0.9211 | 1.097 | 0.9064 |
|  | CRP | 1.002 | 0.999 | 1.005 | 0.199 |
|  | PCT | 1.002 | 0.9966 | 1.006 | 0.546 |
|  | Noradrenaline | 0.9876 | 0.9697 | 1.006 | 0.1834 |
|  |  |  |  |  |  |

OR: Odds Ratio, HR: Hazard Ratio
